# A specific reconditioning training program implemented 12 months after ACL surgery improves lower-limb jump variables in amateur soccer players

**DOI:** 10.3389/fphys.2025.1630156

**Published:** 2025-08-05

**Authors:** Sergio Jiménez-Rubio, Tomás García-Calvo, Luis Manuel Martínez-Aranda, Javier Raya-González

**Affiliations:** ^1^ Sport Sciences Research Centre, Rey Juan Carlos University, Madrid, Spain; ^2^ Faculty of Sport Sciences, University of Extremadura, Cáceres, Spain; ^3^ Department of Sports and Computer Sciences, Faculty of Sports Sciences, Universidad Pablo de Olavide, Seville, Spain; ^4^ Science-Based Training Research Group (SEJ-680), Physical Performance and Sports Research Center, Universidad Pablo de Olavide, Seville, Spain; ^5^ Research Group on Sport and Physical Education for Personal and Social Development (GIDEPSO), Department of Specific Didactics, Faculty of Education Sciences and Psychology, University of Córdoba, Córdoba, Spain

**Keywords:** anterior cruciate ligament, football, neuromuscular training, return to play, recovery

## Abstract

**Introduction:**

Introduction Soccer players are frequently exposed to high physical demands, which increase their risk of injury, especially anterior cruciate ligament (ACL) ruptures. Despite completing standard rehabilitation protocols, many athletes show persistent functional deficits one year after surgery. This study aimed to assess the impact of a 12-week reconditioning training program, focusing on adjacent joint mobility, neuromuscular control, plyometrics, stability-landing exercises, and strength production, of amateur soccer players 12 months after undergoing anterior cruciate ligament (ACL) surgery.

**Methods:**

Twenty-five Spanish male amateur soccer players (age = 21.2 ± 2.4 years) participated voluntarily. All participants followed similar return-to-play guidelines and were randomly assigned to either the control group (CG, n = 13) or the experimental group (EG, n = 12). Soccer players in the EG underwent the 12-week training program (ACLrPRO), and both groups completed jump battery tests before and after the intervention period.

**Results and discussion:**

The results showed improvements in all analyzed variables for the EG, except for the time to stabilization during the single-leg land and hold test for their non-injured leg, where the CG showed better results. Moreover, players in the CG exhibited a decline in performance related to their non-injured leg across all performed tests. The EG also demonstrated lower absence days during the experimental period compared to the CG. These findings underscore the significance of implementing a targeted neuromuscular training program for soccer players recovering from an ACL rupture, especially in enhancing performance and reducing absence days after their return to competition.

## 1 Introduction

Soccer is a demanding team sport characterized by a significant number of high-intensity actions, such as jumping or sprinting (Gualtieri et al., 2023). In recent years, there has been an increase in the occurrence of these high-intensity actions during soccer matches (Allen et al., 2023; Lago-Peñas et al., 2023), prompting notable changes in training methods. The main aim of this is to ensure that soccer players are well-prepared to meet these higher demands (Castillo et al., 2021) and increase the chances of success (Faude et al., 2012). However, these heightened demands are associated with an increased risk of injury in soccer players (Gabbett, 2016), making injuries a primary concern for strength and conditioning coaches in soccer (Raya-González et al., 2019). This is critical as injuries negatively impact both individual player performance (Raya-González et al., 2022) and the collective performance of the team (Hägglund et al., 2013). Moreover, injuries have adverse effects on financial aspects of clubs (Ekstrand, 2013) and on the quality of life of soccer players, sometimes leading to withdrawal from competition (Øiestad et al., 2018). These negative effects are particularly pronounced in the case of long-term injuries, with ligamentous injuries, especially ruptures of the anterior cruciate ligament (ACL) of the knee, standing out prominently.

Overall, injury incidence in professional male soccer players is stablished at 8.1 injuries/1,000 h of exposure, with a higher injury incidence during matches (36 injuries/1,000 h of exposure) compared to training sessions (3.7 injuries/1,000 h of exposure) (López-Valenciano et al., 2019). Specifically, joint injuries present an incidence of 0.4 injuries/1,000 h of exposure, with the most common ones related to knee and ankle joints having values close to 1.0 joint injury/1,000 h of exposure (López-Valenciano et al., 2019). Regarding to the ACL knee injuries, Requejo-Herrero et al. (2023) obtained an incidence of 0.0364 ACL injuries/1,000 h of exposure in professional male soccer players participating in the Spanish first division. Similarly, an incidence of 0.076 ACL injuries/1,000 h of exposure in were observed by Rekik et al. (2018) in male soccer players belonging to a Middle Eastern league. Although the incidence of ACL injuries in male soccer players is lower compared to other types of injuries, such as muscle-tendon injuries where the hamstring complex is involved (Diemer et al., 2021), the severity of ACL injuries is high. In this regard, Requejo-Herrero et al. (2023) and Rekik et al. (2018) revealed that the mean lay-off time after an ACL injury is close to 220 absence days. Given that the absence days associated with these injuries are very high, their negative impact on soccer players is excessively significant, sometimes even leading to retirement. Therefore, it seems essential to understand their impact on players and establish appropriate strategies to alleviate these effects.

After an ACL injury and subsequent surgery, it has been observed that the mechanoreceptors of the knee fail to send information for neuromuscular responses to maintain balance and joint function. This results in certain instability of the postural control system and muscle strength deficits (Negahban et al., 2014), accompanied by a decrease in quadriceps activation and increased flexor muscle activity of the knee (Nyland et al., 2016). In this context, the primary objective of rehabilitation programs for ACL injuries is to enhance the strength level, particularly of the knee extensor muscles (Buckthorpe et al., 2019). This improvement is crucial before restoring the quality of movement, functional strength, power, explosive muscle strength, and undertaking sport-specific retraining for the eventual return to sport (Buckthorpe and Roi, 2017). Consequently, resistance training becomes a pivotal component in rehabilitation programs after an ACL injury. However, a recent systematic review conducted by Nichols et al. (2021) has revealed a misalignment between the intensity prescribed during resistance training programs for ACL rehabilitation and strength and conditioning principles. Often, these programs are not optimized to develop the necessary neuromuscular qualities required for return-to-sport criteria. This contributes to suboptimal prescription of rehabilitation programs, resulting in athletes who are inadequately prepared. In this sense, Khalid et al. (2022) concluded that, compared to traditional resistance training, neuromuscular training was significantly more effective in reducing pain, improving function, quality of life, strength, and power. Current studies have validated systems for the quantification of variables related to pathologies that cause deficits in strength, stability and movement imbalance (Collings et al., 2024). Therefore, it is essential to establish rehabilitation programs that facilitate achieving the appropriate values for a safe return to sport.

Return to sport criteria following an ACL injury have been developed to objectively determine when a safe return to sport is indicated (Kyritsis et al., 2016). Regarding this, there is a consensus that passing the objective return to sport criteria is vital in reducing injury risk following an ACL injury (Losciale et al., 2019). Specifically, Kyritsis et al. (2016) found a fourfold increased risk of ACL rupture for those athletes who did not pass the return to sport criteria. Similarly, Grindem et al. (2016) estimated an 84% lower knee reinjury rate among athletes who passed return to sport criteria. However, despite compliance with established guidelines, some authors have reported functional deficits in athletes after returning to sports practice (Nagai et al., 2020). Biau et al. (2007) revealed that only 33% of ACL surgically operated patients with hamstring graft and 41% with patella graft returned to their pre-injury athletic condition, increasing the risk of reinjury. Current trends suggest extending the return to play (RTP) period to 12 months. However, a recent study (Felix et al., 2022) demonstrated that functional outcomes increased in athletes with ACL reconstruction after 12 months, but did not reach the same level as the control group. These authors found incomplete functional recovery and adaptive changes in postural control after injury, reconstruction, and return to sport. Considering this, it seems essential to apply neuromuscular programs in the 12 months following ACL surgery, even if the soccer player has satisfactorily returned to sport.

Thus, the aim of this study was to evaluate the effects of a specific reconditioning program training based on adjacent joint mobility, neuromuscular control, plyometrics, stability-landing exercises, and strength production exercises on the performance and availability of amateur soccer players applied 12 weeks after an ACL surgery. Based on prior studies (Khalid et al., 2022; Zsidai et al., 2023), we hypothesized that players in the experimental group would improve their performance related to biomechanical jump variables and would present lower absence days during the experimental period.

## 2 Materials and methods

### 2.1 Study design

A randomized-controlled trial design was applied to investigate the effects of a multicomponent training program, comprising adjacent joint mobility, neuromuscular control, plyometrics, stability-landing exercises, and strength production exercises, on the performance and availability of amateur soccer players. This training program was implemented 12 months after an ACL surgery and lasted 12 weeks (i.e., three sessions per week). Prior an after the experimental period, participants performed the following jump tests: single leg land and hold (SLLH); single leg jump (SLJ); single leg squat assessment (SLSA); drop jump (DJ), considering several biomechanical variables for each test. These jump tests were performed with both, injured and non-injured legs, Additionally, the number of absence days from regular soccer training during the experimental period was registered. Jump tests were conducted in a single session in the morning (9–11 a.m.) in a controlled indoor environment with temperatures maintained between 20°C and 23°C and humidity levels between 60% and 70%. The assessment sessions were supervised by the same strength and conditioning specialist and a standardized warm-up was completed by participants. Players were instructed to have their last meal at least 2 h before the start of the tests and to avoid consuming caffeinated beverages.

### 2.2 Participants

Twenty-five Spanish male amateur soccer players (age = 21.2 ± 2.4 years) voluntarily participated in the study. An *a priori* power analysis was performed (G*Power, v3.1.9.2, Universität Kiel, Germany), which determined that a sample size of at least 20 participants was needed to achieve a power (1-β) of 0.84, assuming an effect size (ES) of 0.90 (large effect) and alpha of 0.05. Participants belonged to different teams competing in semiprofessional league of Spain, and trained 4 times per week and played one official match during the weekend. Also, players covered the satisfactorily similar RTP guidelines prior to be allocated in any group. The inclusion criteria for the study were as follows: amateur soccer players over 18 years of age with no history of cardiovascular or metabolic pathologies, with 12 months postoperative anterior cruciate ligament injury, with the capacity to perform stability drills on one leg and without injuries during the research process. Players with 2 or more joint injuries in the 3 months prior to the investigation and with a recent history (i.e., less than 4 months) of knee surgery were excluded from the study. Participants were randomly assigned to either the control group (CG; n = 13; age: 20.2 ± 1.9 years; height: 179.0 ± 0.1 cm; body mass: 70.3 ± 2.3 kg; body mass index: 21.9 ± 0.8 kg·m−2) or to the experimental group (EG; n = 12 age: 22.2 ± 2.5 years; height: 180.0 ± 0.1 cm body mass: 70.8 ± 2.6 kg; body mass index: 21.8 ± 0.6 kg·m−2).

### 2.3 Procedures

During the 12-weeks intervention period, players performed their regular weekly in-season routine, with the EG performing Anterior Cruciate Ligament Reconditioning Program (ACLrPRO) 3 times per week, in addition to their regular soccer training routines. All participants were familiar with the testing protocols. Prior to the experimental sessions, players undertook a 12-min standardized warm-up consisting in 4 blocks: a) myofascial inhibition, participants used foam roller and pressure balls to facilitate the myofascial level in quadriceps, soleus and hip extensors; b) mobility, facilitated joint range of motion of the thoracic spine, ankle dorsiflexion, hip in submaximal range of flexion combined with external rotation, overhead squat and analytical facilitation of internal and external hip rotation; c) muscle activation, based on the application of tensions to the posterior chain with three exercises that combined isometric and short cycles of stretching-shortening in safe levers; and d) adaptation to stretch-shortening cycle, applying the single leg jump exercise with take-offs of only 2 cm above the surface and the countermovement jump without seeking maximum height, but rather motor control in the deceleration part.

#### 2.3.1 Instrument

All test were completed on 2 portable force platforms (FD4000 ForceDecks dual force platforms; VALD Performance, Sydney, Australia) and neuromuscular performance techniques ForceDeck Software (version 1) at a sampling frequency of 1,000 Hz. This software detects the initiation of movement as a 30 N deviation from the initial bodyweight calculation, eccentric to concentric phase moment as the lowest center of mass displacement, and take-off as the moment the vertical forces fall 30 N below body mass. A series of metrics from ForceDecks software’s default output were analyzed to provide the end user with reliability of a multitude of metrics of interest. These metrics are defined elsewhere (Chavda et al., 2018; Heishman et al., 2020; Merrigan et al., 2020) and can also be found in the ForceDecks user guide. Raw data were analyzed via custom-designed Microsoft Excel Software (Redmond, WA) and filtered of high-frequency noise using a Butterworth low pass filter at 10 Hz.

#### 2.3.2 Jump tests

Single Leg Land and Hold (SLLH). Players conducted four attempts with each leg, separated by 30 s of passive recovery between the two legs but continuously between repetitions of the same leg. The 2 first attempts were used to assess the Peak Drop Landing Force (PDLF) variable, both for the uninjured leg (PDLFnon) and the injured leg (PDLFinj). To measure this, participants initially weighed themselves with both feet on the platforms and then stood on a 40 cm high box. While on the box, participants placed the toes of the standing leg on the red line marked at the edge of the box. From this starting position, participants landed safely on the corresponding platform to record the peak force during this unilateral landing. To evaluate the Time to Stabilization (TSTAB) variable for both the uninjured leg (TTSnon) and the injured leg (TTSinj), participants completed other two attempts of the SLLH as follow: the participants weighed themselves with both feet on the platforms, stepped off the platforms to initiate the evaluation, and then, from a line marked 15 cm from the base of each platform, performed a single-leg landing. The time required to stabilize in a triple flexion pattern without destabilizing the platform was quantified. The ICCs and the CVs for these variables were 0.73–0.81 and 3.3–4.4, respectively.

Single Leg Jump (SLJ). Players performed two attempts with each leg, with 30 s of passive recovery between the two legs but continuous repetitions with the same leg. Firstly, the participants weighed themselves with both feet on the platforms. Then, each participant stabilized on the non-injured leg and performed a single leg squat with a stretch-shortening cycle. This was followed by a jump-push off to take off as much as possible on that platform. After finishing these 2 executions with the healthy limb, the same procedure was done with the injured limb, without the need for weighing and without modifying the registration in the software. Derived from this test, Jump Height (JH) and Peak Power Related to Body Mass (PPOW) were obtained, selecting the highest value in cm and W/kg, respectively. The ICCs and the CVs for JH were 0.64–0.78 and 5.5–5.7 respectively, while for PPOW were 0.70–0.79 and 2.2–2.5 respectively.

Single Leg Squat Assessment (SLSA). Players completed two attempts with each leg, with a 30-s passive recovery between the two legs but continuous repetitions with the same leg. After the weight measurement, with the participant in a bipodal stance, we recorded their ability to generate the maximum force in a single-legged squat without take-off. This process was repeated twice, starting with the non-injured limb and then repeating the same process to measure the maximum force produced with the injured limb. The highest values records in N for each limb was used to determine the Peak Push Force (PPFORCE). The ICCs and the CVs for this variable were 0.69–0.75 and 3.8–3.9, respectively.

Drop Jump (DJ). Players completed two bilateral DJ attempts, with a 1-min passive recovery period between them. Participants were instructed to stand in an upright position on the 30 cm box, with their feet shoulder-width apart and hands placed on their hips. Then, they were instructed to step off the box, descend to land evenly on both feet (with no heel contact allowed), and immediately perform a maximal-effort vertical jump. The best jump was selected to calculate the Reactive Strength Index (RSI) using the ratio of jump height to contact time. The ICCs and the CVs for RSI were 0.70 and 4.4 respectively.

#### 2.3.3 Absence days

During the 12-weeks intervention period, the days on which players could not participate in the regular training soccer sessions of their team due to pain, discomfort or insecurity related to the injured leg were registered.

#### 2.3.4 ACLrPro multicomponent training program

The training intervention lasted 12 weeks, with 3 sessions per week. The ACLrPRO incorporated mobility tasks of the ankle, hip, thoracic spine and stability in load, primarily in unilateral scenarios, implementing landings, strength and neuromuscular control drills, explosive and reactive tasks in the absorption-transmission relationship, as well as tasks emphasizing multiplanar force production. Players were introduced to the ACLrPRO exercises 1 year after surgery and only if players had satisfactorily completed the return to competition. Each training session lasted between 20 and 55 min and took place after the standardized warm-up previously explained. The ACLrPRO sessions took place four (MD-4), three (MD-3) and one (MD-1) days prior the matches. The ACLrPro sessions were supervised by a qualified strength and conditioning coach, who provided adequate feedback and instruction for correctly executing the drills. To ensure progressive improvement, a progressive overload approach was implemented, tailoring the workload for each drill based on individual capabilities (Table 1).

**TABLE 1:** Anterior Cruciate Ligament Reconditioning Program (ACLrPRO) applied in the experimental group.

Weeks	Loading focus-target	Aims	Contents	Progression criteria in this structure	Neuromuscular load (TUT-drill)
0–2	Mobility Neuromuscular Control Strength (ISO, assisted with not injured limb, SSCi slow velocity, plyo low impact one legged)	To facilitate dorsiflexion of ankle, rotations hip, extension thoracic spine, mobility first toe and subtalar injury leg and central structure stability To increase one-legged stability capacity, and generate superior levels of neuromuscular control in previous injured leg as well	Drills of mobility as joint by joint concept Single leg assisted drills	Time and complexity in mobility drills under load Progress with initiation of one-legged stability tasks with minimal ground clearance on stable surfaces	10 tasks: 2 × 8″ each MD-4/MD-3 6 tasks: 2 × 10″ each MD-1
3–4	Mobility Neuromuscular Control Strength (ISO, assisted with not injured limb SSCi at slowandfast velocity, plyo medium impact one legged, absorption&RFD)	To continue increasing one-legged stability capacity, and generate optimal levels of neuromuscular control on one leg as well To implement strength levels and neuromuscular control in the posterior chain (glute, hamstrings and soleus)	Single leg assisted squat Single leg squat on box: Iso + SSC with time under tension about 20 s for drill. Landing drills, vertical and horizontal stimulus Horizontal Force drills Absorption forces on leg from to box 40 cm	Increased times during the Stretch-shortening cycle to reach drills with 25–30 s of time under tension. Increased complexity in landing drills, combining force absorption and force transmission actions.	3 tasks: 5x (4”+PUSH-CONC) MD-4/MD-3 3 tasks: CSTC- 3x (10”+6”+4”+1″) MD-4/MD-3 2 tasks: Horizontal Force 2x (15”+6PR+4hip ABDs)
5–7	Mobility Neuromuscular Control with perturbations in high boxes Strength (ISO, assisted with not injured limb SSCi slowandfast velocity, plyo high impact one legged, absorption&RFD, push ground in the finish of the drills for COD)	To train strength manifestations of the one-legged stretching cycle and agility capabilities through reactivity in linear displacements To increase movement skills to optimize athlete movement agility	Wall drills Plyo training bi and one leg Iso + SSC + Plyo training with drills with 20–25 s of time under tension. Rate force development in actions that combined landing and push for acceleration	Eccentric strength exercises with absorption contact in less conscious time than previous drills to change plane and reduce contact times. Combination of one-legged jumping drills on a 30–40 cm box and performance followed by sled pushing interventions over 15 m stretches in 4–5 s with a load of 40%–50% of body weight.	3 tasks: CSTC- 2x (8”+8”+6”+2″) MD-4/MD-3 4 tasks: CSTC- 3x (10”+8”+8”+1″) MD-4/MD-3 2 tasks: CSTC- 2x (8”+8”+8”+1″) MD-1 3 tasks: PLYO ONE LEG (30–40 cm) 15–20 s for drill MD-3/MD-1
8–10	Reactive force and ability to stabilize and push the ground in different planes after stabilization	To train the ability to jump on one leg with emphasis on the absorption-production cycle of force so that it occurs in the shortest possible time and generates the greatest possible height To train strength manifestations of the one-legged stretching cycle and agility capabilities through reactivity in multidirectional displacements	Plyo one leg + COD training Acc-Decc patterns + multidirectional skills Agility abilities with previous single leg squat and deadlift drills Elastic explosive reflex manifestation of one-legged strength	Drills for the improvement of vertical and horizontal explosiveness, with very reactive jumps to the movement in any direction. Increased height and decreased contact times followed by reactive accelerations in the sagittal frontal and transverse planes	3 tasks: CSTC- 2x (8”+8”+6”+2″) MD-4/MD-3 3 tasks: CSTC- 3x (10”+8”+8”+1″) MD-4/MD-3/MD-1 3 tasks: PLYO ONE LEG (30–40 cm) 18–25 s for drill MD-3/MD-1
11–12	Reactivity, explosive force and multiplanar stability-push complex	To Increase explosive strength, reactivity and the ability to decrease the time between stabilization and explosive thrust against the ground to change plane with uncertainty	Plyo one leg + COD training Acc-Decc patterns + multidirectional skills Agility abilities with previous single leg squat and deadlift drills Elastic explosive reflex manifestation of one-legged strength	Drills for the improvement of vertical and horizontal explosiveness, with very reactive jumps to the movement in any direction. Increased height and decreased contact times followed by reactive accelerations in the sagittal, frontal and transverse planes	3 tasks: PLYO ONE LEG (30–40 cm) 15–20 s for drill Combined tasks of high neuro Muscular load with duration Greater than 50 s per Task. MD-4/MD-3 = 4 Tasks Each training. MD-1 = 2 tasks

Abbreviations: TUT, time under tension; SSC, stretching short cycle; SSCi, Incomplete Streching Short Cycle; Plyo = Pliometric; COD, change of direction; RFD, rate force development; PUSH-CONC, concentric push on the ground; EQI, Eccentric quasi-isometric; PR, pelvic retroversion; ABDs, Abductions; MD, match day; Iso = Isometric Neuromuscular Load Injured Limb; CSTC , Cycles of Strength Training Concept: (1) time of isometric tension + (2) time of incomplete SSC+ (3) time of complete SSC+ (4) time of SSC, with Push and Absorption forces.

### 2.4 Statistical analysis

Descriptive statistics are presented as mean ± standard deviation (SD). The Shapiro-Wilk and Levene tests were used to prove the normality of the distribution and the homogeneity of variances, respectively. A Paired-samples t-test was applied to analyze within-group differences, while the possible between-group differences were studied through an analysis of covariance (ANCOVA), including baseline values as covariates. Effect sizes (ES) were calculated using Cohen’s ES to assess the magnitude of the effects. ES were interpreted as follow: <0.2, trivial; 0.20 to 0.49, small; 0.50 to 0.80, moderate and >0.80, large (Cohen, 1988). The data analysis was carried out using the Jeffrey’s Amazing Statistics Program (JASP 0.18.1; The JASP team, Amsterdam, The Netherlands) being the statistical significance was set at p < 0.05.

## 3 Results

Table 2 shows the changes observed in the force platform variables related to jump after the intervention period in both groups. Within-group analysis presented significant changes in PDLFnon (*p* = 0.003; ES = −0.51, moderate), TSTABnon (*p* = 0.0042; ES = 0.67, moderate), JHnon (*p* = 0.001; ES = −1.07, large), PPFORCEnon (*p* = 0.001; ES = −1.62, large) and RSI (*p* = 0.002; ES = −1.20, large) for CG, while participants in EG experienced significant changes in all variables (*p* = 0.001; ES = −8.94 to 9.00, large) except for TSTABnon. Between-groups analysis revealed differences in favor of the EG in all variables (F = 7.10 to 81.62; *p* = 0.001) except for TSTABnon.

**TABLE 2:** Changes in biomechanical variables before (baseline) and after (post-training) the 12-week intervention period.

Tests	Variables		CG (n = 13)					EG (n = 12)					Between group differences	
			Baseline (mean ± SD)	Post training (mean ± SD)	Δ (%)	p	ES	Baseline (mean ± SD)	Post training (mean ± SD)	Δ (%)	p	ES	F	p
SLLH	PDLF (N)	PDLFinj	2,574.97 ± 191.37	2,546.21 ± 160.47	−1.13	0.063	−0.15	2,649.14 ± 132.04	2,128.49 ± 68.13	−24.46	**0.001**	−3.93	81.62	**0.001**
		PDLFnon	1864.89 ± 127.08	1799.78 ± 79.92	−3.62	**0.003**	−0.51	1946.46 ± 76.90	1,258.69 ± 135.75	−54.64	**0.001**	−8.94	8.47	**0.001**
	TSTAB (s)	TSTABinj	0.99 ± 0.13	1.02 ± 0.13	2.94	0.395	0.23	1.01 ± 0.05	0.74 ± 0.05	−36.49	**0.001**	−5.40	9.94	**0.001**
		TSTABnon	0.97 ± 0.09	1.03 ± 0.07	5.83	**0.042**	0.67	1.05 ± 0.04	1.06 ± 0.06	0.94	0.696	0.25	0.10	0.784
SLJ	JH (cm)	JHinj	3.86 ± 0.43	3.79 ± 0.17	−1.85	0.572	−0.16	4.01 ± 0.13	5.18 ± 0.14	22.59	**0.001**	9.00	96.43	**0.001**
		JHnon	4.24 ± 0.68	3.51 ± 0.31	−20.79	**0.001**	−1.07	4.26 ± 0.13	5.04 ± 0.24	15.48	**0.001**	6.00	7.10	**0.001**
	PPOW (W/kg)	PPOWinj	16.29 ± 0.62	16.49 ± 0.43	1.21	0.360	0.32	16.27 ± 0.45	18.27 ± 0.32	10.95	**0.001**	4.44	29.54	**0.001**
		PPOWnon	16.43 ± 0.51	16.16 ± 0.48	−1.67	0.095	−0.53	16.50 ± 0.41	19.01 ± 0.42	13.20	**0.001**	6.12	52.18	**0.001**
SLSA	PPFORCE (N)	PPFORCEinj	1,040.15 ± 40.57	1,034.01 ± 41.38	−0.59	0.465	−0.15	1,006.53 ± 51.28	1,269.60 ± 36.64	20.72	**0.001**	5.13	7.51	**0.001**
		PPFORCEnon	1,161.58 ± 65.01	1,055.88 ± 59.62	−10.01	**0.001**	−1.62	1,057.10 ± 42.84	1,219.04 ± 16.31	13.28	**0.001**	3.78	7.30	**0.001**
DJ	RSI (m/s)		0.77 ± 0.05	0.71 ± 0.03	−8.45	**0.002**	−1.2	0.75 ± 0.03	0.96 ± 0.02	21.88	**0.001**	7.00	61.34	**0.001**

Abbreviations: CG, control group; EG, experimental group; SD, standard deviation; Δ (%): percentage of change between pre and post intervention; p: level of significance; ES: effect size; SLLH, single leg land and hold; SLJ, single leg jump; SLSA, single leg assessment; DJ, drop jump; inj = injured leg; non = non-injured leg; PDLF, peak drop landing force; TSTAB, time to stabilization; JH, jump height; PPOW, peak power related to body mass; PPFORCE, peak push force; RSI, reactive strength index.

Bold indicate the p-values that are statistically significant.

A total of 151 absence days (11.6 ± 9.7 per player) were collected for CG during the experimental period, while for the E.G., 64 absence days (5.3 ± 6.1 per player) were recorded in the same period.

## 4 Discussion

This study aimed to evaluate the effects of a specific reconditioning training program based on adjacent joint mobility, neuromuscular control, plyometrics, stability-landing exercises, and strength production exercises on the performance and availability of amateur soccer players applied 12 months after an ACL surgery. Despite several studies have analyzed the effectiveness of several rehabilitation programs after an ACL injury, to date, this is the first study that intervenes on the period after the return to sport, when players are fully incorporated into soccer practice. Following the program intervention, players in the EG improved all analyzed variables except for TSTABnon, obtaining better results compared to the participants in the CG. Additionally, the EG exhibited lower absence days during the experimental period compared to the CG.

Landing ability has been identified as a determinant of injury risk related to ACL injuries (Ohji et al., 2021), justifying its inclusion as a crucial component in preventive programs for this injury. However, there are several variables implicated in landing that must be considered. In this regard, PDLF holds great relevance, as reducing the impact forces during landing after an ACL injury is essential to decrease the risk of reinjury (Bressel and Cronin, 2005). In this study, significant decreases in PDLFinj and PDLFnon were observed, with better results compared to the CG, which also show a decrement in PDLFnon. These improvements, particularly in the injured leg, could be attributed to the high unilateral training load developed during the intervention period (Oliveira et al., 2022). This training program was focused on the specific needs of the injured leg, addressing aspects such as reception technique, strength, and stability. Another relevant variable during landing is the TSTAB, which measures the time it takes for an individual to return to a baseline or stable state following a jump or hop landing (Fransz et al., 2015). An increase in TSTAB, particularly related to an ACL injury, indicates dynamic postural control deficits, reflecting difficulties in controlling ground reaction forces (Webster and Gribble, 2010). Our results align with those previously obtained by Kasmi et al. (2021), who observed reductions in TSTAB after implementing a training program based on eccentric and plyometric exercises 14 weeks after ACL surgery in elite female athletes. Specifically, in our study, players in the EG improved their TSTAB in the injured leg after the experimental period. The inclusion of tasks based on the stretching-shortening cycle, emphasizing minimizing ground contact time, could explain this improvement. Conversely, no improvements in the non-injured leg were observed, although the program prevented an increase in TSTAB similar to the CG, justifying its value for both the injured and non-injured leg.

Prior studies have observed a decrease in jump performance after ACL surgery in male athletes (King et al., 2018; Kotsifaki, Whiteley, et al., 2022), possibly as a consequence of the neurophysiological alterations in the ACL following the rupture (Kakavas et al., 2020). In this regard, the SLJ test appears to be a key tool for identifying knee function deficits upon return to sport after ACL reconstruction (Kotsifaki, Van Rossom, et al., 2022). This test allows the assessment of various variables such as jump height (JH) and peak power related to body mass (PPOW), providing a comprehensive understanding of the injured leg’s status. In this study, players in the EG demonstrated improvements in both variables after the 12-week intervention period. This is particularly relevant since soccer players not only enhanced the power exerted but also exhibited improved neuromuscular performance, as evidenced by increased strength and coordination during the jump, resulting in greater jump height. Such improvements may be associated with a lower risk of reinjury, given previous findings indicating that soccer players, even though meeting RTP criteria, fail to fully regain pre-injury physical fitness values 12 months post-operation (Felix et al., 2022). Additionally, soccer players in the EG showed enhanced performance in both variables in the non-injured leg. This suggests that the applied program could significantly influence players’ overall sports performance (Zahalka et al., 2021). Conversely, players in the CG did not show improvements in either variable and, notably, experienced a significant reduction in jump height on the non-injured leg. These results provide evidence supporting the effectiveness of the program in better preparing soccer players for competition compared to those who did not undergo the intervention, despite all players having satisfactorily complied with RTP guidelines and actively participated with their teams.

The SLSA test assesses peak push force (PPFORCE) during a squat, which is closely related to the force exerted by the knee extensor apparatus in athletes who suffer an ACL rupture (Batty et al., 2019), essential during the rehabilitation process (Buckthorpe et al., 2019). After the intervention, players in the EG experienced a significant increase in the PPFORCE in their injured leg, nearly 20%, equating the PPFORCE levels to those of the uninjured leg, which also improved its performance (i.e., 13%). This finding is highly relevant, as a great force level of the knee extensors is the basis for other critical physical qualities such as quality of movement, functional strength, or power, facilitating an adequate level of physical fitness and favoring a reduced reinjury risk (Buckthorpe and Roi, 2017). Similar to JH, players in the CG worsened their performance relative to the PPFORCE in their non-injured leg, showing lower values in both legs compared to their EG counterparts after the 12-week intervention period. Some authors have reported that substantial deficits in plyometric abilities (e.g., reactive strength) are common after ACL reconstructions (Read et al., 2023). Therefore, the reactive strength index (RSI) measured during a DJ seems to be a key variable to consider when players return to play (RTP) after an ACL injury (Jarvis et al., 2022). In this sense, RSI provides a valuation of knee function regarding the stretch shortening cycle, as this metric refers to the ability of the muscle-tendon units to produce a rapid and powerful contraction immediately following a rapid eccentric action (Ramirez-Campillo et al., 2018). Improvements in RSI were observed in the EG after the application of the ACLrPRO, possibly due to a significant change in neuromuscular control and the ability to produce force. This implies an increase in the ability to apply force at high velocity through the combination of musculotendinous elasticity and contractility in a task such as the drop jump. Similar to prior variables, RSI performance also decreased in the CG, suggesting the need to implement neuromuscular training programs 12 months after ACL surgery to avoid reductions in performance that may lead to reinjuries or new associated injuries.

Previous studies have highlighted limitations in the return to play and performance, with a 15-year follow-up registering up to 35% of participants facing restrictions in returning to professional soccer (Waldén et al., 2016). Similarly, other studies with a 6-month post-surgery ACL follow-up reported 24% of participants experiencing relapses in the process. However, in this same study, it was observed that by maintaining tissue maturation until 2 years, 80.7% of participants could secure a return to play (Bodkin et al., 2022). Therefore, it appears pertinent to document the days on which players could not participate in regular soccer training sessions with their team due to pain, discomfort, or insecurity related to the injured leg to gather comprehensive information about the effectiveness of the ACLrPRO. In this context, players in the CG group experienced 151 absence days (11.6 ± 9.7 per player) during the 12-week period, while only 64 absence days (5.3 ± 6.1 per player) were recorded for players in the EG during the same period. This finding supports the assertion made by Felix et al. (2022), which claimed that soccer players exhibit incomplete functional recovery and adaptive changes in postural control after injury, reconstruction, and return to sport after 12 months of ACL reconstruction. It confirms the necessity of implementing a neuromuscular training program in this population, not only to enhance performance and reduce the reinjury risk but also to increase the availability of soccer players after their return to competition.

This study has some limitations that practitioners should be aware of. The primary limitation is the inability to control the workload covered by soccer players in their weekly microcycles with their respective clubs during the intervention period. Additionally, the sample size (n = 25) could be scarce to stablish robust conclusions. However, the analyzed topic (LCA injuries), with high number of absence days, difficult to involve a higher sample. Also, the intervention involved only male soccer players, so caution should be exercised when generalizing the findings to women’s soccer, considering the differences between the groups in terms of physical, physiological, and biomechanical factors. Finally, there was no follow-up after the 12-week intervention. Future studies should consider long-term follow-up, such as one and 2 years after completing the program, to assess the status of soccer players, the incidence of reinjuries, and possible player withdrawals.

## 5 Conclusion

In summary, players in the EG improved all analyzed variables except for TSTABnon, obtaining better results compared to their counterparts in the CG after performing the ACLrPRO. Additionally, players in the CG decreased their performance related to their non-injured leg in PDLF, TSTAB, JH, PPFORCE, and RSI. Finally, the EG exhibited lower absence days during the experimental period compared to the CG. These findings suggest the importance of implementing a neuromuscular training program in soccer players who suffered an ACL rupture after they returned to competition, not only to enhance their performance, reduce the reinjury risk, and increase their availability but also to avoid a decrease in performance related to the non-injured leg.

## Data Availability

The original contributions presented in the study are included in the article/supplementary material, further inquiries can be directed to the corresponding author.
